# Gut Microbiome of Indonesian Adults Associated with Obesity and Type 2 Diabetes: A Cross-Sectional Study in an Asian City, Yogyakarta

**DOI:** 10.3390/microorganisms9050897

**Published:** 2021-04-22

**Authors:** Phatthanaphong Therdtatha, Yayi Song, Masaru Tanaka, Mariyatun Mariyatun, Maisaroh Almunifah, Nancy Eka Putri Manurung, Siska Indriarsih, Yi Lu, Koji Nagata, Katsuya Fukami, Tetsuo Ikeda, Yuan-Kun Lee, Endang Sutriswati Rahayu, Jiro Nakayama

**Affiliations:** 1 Department of Bioscience and Biotechnology, Faculty of Agriculture, Kyushu University, 744 Motooka, Nishi-ku, Fukuoka 819-0395, Japan; vo_21851@hotmail.com (P.T.); lana745143663@gmail.com (Y.S.); msr456852@gmail.com (M.T.); 2 Faculty of Agricultural Technology, Universitas Gadjah Mada, Yogyakarta 55281, Indonesia; maria_slimshady@yahoo.com (M.M.); almunifah@gmail.com (M.A.); nancyekaputri@gmail.com (N.E.P.M.); siskaindriarsih@gmail.com (S.I.); endangsrahayu@ugm.ac.id (E.S.R.); 3 Department of Applied Biological Chemistry, Graduate School of Agricultural and Life Sciences, The University of Tokyo, 1-1-1 Yayoi, Bunkyo-ku, Tokyo 113-8657, Japan; ly22999@hotmail.com (Y.L.); aknagata@mail.ecc.u-tokyo.ac.jp (K.N.); 4 Material Management Center of Kyushu University, 744 Motooka, Nishi-ku, Fukuoka 819-0395, Japan; kfukami@mmc.kyushu-u.ac.jp; 5 Department of Surgery and Science, Graduate School of Medical Sciences, Kyushu University, 3-1-1 Maidashi, Higashi-ku, Fukuoka 812-8582, Japan; t-ikeda@surg2.med.kyushu-u.ac.jp; 6 Endoscopy and Endoscopic Surgery, Fukuoka Dental College, 2-15-1 Tamura, Sawara-ku, Fukuoka 814-0193, Japan; 7 Department of Microbiology and Immunology, National University of Singapore, 5 Science Drive 2, Singapore 117545, Singapore; micleeyk@nus.edu.sg

**Keywords:** gut microbiome, bile acids, dietary habits, obesity, type 2 diabetes

## Abstract

Indonesia is a developing country facing the national problem of the growing obesity and diabetes in its population due to recent drastic dietary and lifestyle changes. To understand the link between the gut microbiome, diet, and health of Indonesian people, fecal microbiomes and metabolomes of 75 Indonesian adults in Yogyakarta City, including obese people (*n* = 21), type 2 diabetes (T2D) patients (*n* = 25), and the controls (*n* = 29) were characterized together with their dietary and medical records. Variations of microbiomes showed a triangular distribution in the principal component analysis, driven by three dominant bacterial genera, namely *Bacteroides*, *Prevotella*, and *Romboutsia*. The *Romboutsia*-driven microbiome, characterized by low bacterial diversity and high primary bile acids, was associated with fat-driven obesity. The *Bacteroides*-driven microbiome, which counteracted *Prevotella* but was associated with Ruminococcaceae concomitantly increased with high-carbohydrate diets, showed positive correlation with T2D indices but negative correlation with body mass index. Notably, *Bacteroides fragilis* was increased in T2D patients with a decrease in fecal conjugated bile acids, particularly tauroursodeoxycholic acid (TUDCA), a farnesoid X receptor (FXR) antagonist with anti-diabetic activity, while these features disappeared in patients administered metformin. These results indicate that the gut microbiome status of Indonesian adults is differently associated with obesity and T2D under their varied dietary habits.

## 1. Introduction

The Asian microbiome project (AMP) was established in 2009 with the aim of investigating the links between different traditional diets, gut microbiome, and health. Thus far, the AMP has conducted three phases of research in 10 countries [1,2,3] (http://www.agr.kyushu-u.ac.jp/lab/microbt/AMP/: accessed 3 March 2021). These outcomes suggest that modernization occurring in Asian countries is remodeling the gut microbiome of Asians with dietary changes. Therefore, a question arises as to how the remodeled gut microbiome affects the health of Asian people. To answer this question, the AMP phase IV is conducted with the aim of focusing on obesity and diabetes as lifestyle diseases, most probably sensitized by dietary change.

Since gut microbes interact with host immune and hormonal systems via cell components or metabolites, alterations of the gut microbiome and its function may be crucially involved in metabolic disorders, such as obesity and T2D [4,5]. To address this notion, many studies have attempted to identify microbiome features associated with the development of these diseases. In previous studies, although the gut microbiome of obese individuals mostly expressed low bacterial diversity reflecting the gut dysbiosis [6,7], T2D individuals showed variable results [8,9] suggesting external complex factors, including drug intake, host genetic factors, and their surrounding environmental factors, including changing dietary habits [9,10].

Gut bacteria digest complex carbohydrates and fermentatively produce short chain fatty acids (SCFA) and intermediate metabolites, such as lactate and succinate [11]. Since it is known that these products are directly or indirectly involved in metabolic and energy homeostasis [12,13], it is believed that dysfunction of their biosynthesis is linked to metabolic diseases. In addition, much attention has been paid to bile acids (BA), which are synthesized by the host but derivatized by gut microbes through deconjugation, dehydroxylation, and epimerization. In addition to their original function as lipid surfactants, BAs have hormonal functions through host receptors, such as FXR [14] and the membrane protein Takeda G protein-coupled receptor 5 (TGR5) [15]. These receptors transmit intestinal BA signals to the liver, thereby regulating host energy and metabolic homeostasis. Since bacterially derived BAs have stronger activity with the receptors, BA metabolism in the intestine is crucial for host homeostasis, and its disorder may lead to metabolic diseases [16]. It is also noted that BAs have antimicrobial effects, which may be involved in the structure of the gut microbiome by providing selective pressure of bile-sensitive bacteria [17].

Obesity in Asia is now catching up with the West due to economic growth in many Asian countries in recent decades. There are factors contributing to the prevalence of this disease, including migration from rural to urban areas and rapid socioeconomic transition, both of which are associated with lifestyle changes among Asians, such as reduced physical activity and intake of an energy-dense diet [18]. Obesity is correlated with T2D via the development of insulin resistance by adipose tissue in the body [19], although not all obese individuals develop T2D, suggesting an anti-T2D mechanism may be present in metabolically normal obese subjects [20,21]. On the other hand, Asians are at a high risk of diabetes even though they are not obese [22,23], while the form of diabetes occurring in the context of obesity [24] in Asia has been increasing gradually. This emerging risk can be explained by three main causes: heredity, physiologies, and dietary consumption behaviors among Asian people [25,26]. In particular, changes in dietary patterns in Asian modernization from a plant-based traditional diet rich in complex carbohydrates to Western-type modern diet rich in animal fat and simple sugars sensitize Asian people to the risk of diabetes [27].

Indonesia is a highly populated country that is a representative of Southeast Asia in terms of lifestyle and diet, notably a rice-based daily diet. The previous study in AMP phase I and phase II indicated that the majority of Indonesian people harbor a gut microbiome highly populated by *Prevotella*, which is predominantly found in people in developing countries or vegetarians [1,28,29]. However, the dietary habits in Indonesia have modernized remarkably in the past quarter-century, which appears to be associated with a dramatic increase in obesity and diabetes populations, accounting for 5.7% and 7.0%, respectively, of Indonesia’s 258 million people in 2016 [30]. Particularly diabetes, Indonesia is the world’s top 10 countries, having a high number of diabetic patients in adults (20–79 years old), accounting for 10.7 million people in 2019 and it has trend to increase to 13.7 and 16.6 million people in 2030 and 2045, respectively [31]. Trend in consumption of calorie-dense diet of Indonesian people affecting their health has been investigated by several studies. An observational study by publicly available data from the Indonesian family life survey during 1993–2014 found that Indonesian people gain more weight, the prevalence of which dramatically increases in both adults and children and is associated with high consumption of ultra-processed foods together with decreasing level of physical activity [32]. People in Yogyakarta City had prevalence of dyslipidemia associated with high consumption of fatty, grilled, and processed foods, and low consumption of fruits and vegetables [33]. Moreover, high rate consumption of snack foods was found in school-age children in rural area of West Java [34].

In this study, a pilot-scale cross-sectional study in Yogyakarta City as a representative of a developing city in Asia was conducted (i) to investigate the association between diet and gut microbiome that affects Indonesian’s health, and (ii) to capture the status of gut microbiome and metabolomes in Indonesian people that links to obesity and T2D.

## 2. Materials and Methods

### 2.1. Ethics Declaration

This study was approved by the Ethics Committees of the Faculty of Agriculture at Kyushu University (No. 17–55) on January 29th, 2018 and Universitas Gadjah Mada (UGM) No.KE/FK/1017/EC/2018) on September 20th, 2018. All methods were carried out in accordance with relevant guidelines and regulations. Written informed consent was obtained from all subjects participating in this study. Samples and questionnaire data were entered and analyzed anonymously and will publish all data anonymously using patient numbers.

### 2.2. Study Design

In this study, only Indonesian adult males who lived in Yogyakarta City were targeted to avoid the effect of micro-genderome, which may contribute to gender bias in the results, notably the effect of postmenopausal hormonal change on the gut microbiome occurring during the ages targeted in this study [35]. Subject screening was performed based on inclusion and exclusion of the study criteria (see Supplementary Materials for more details). Physical and clinical data of the subjects were measured at UGM hospital. Subjects who qualified for the inclusion criteria were further involved in the activity for seven days by filling out the questionnaire, including subjects’ daily notes, medical records, and dietary records. On the 8th day, subjects were asked to collect their fecal samples using the sampling kits provided by the researchers, and they were asked to submit the complete questionnaire. Eventually, 75 subjects were included in this study. The 75 subjects were classified into two groups related to diabetes (T2D and non-T2D) according to FBG value, as well as three groups according to body mass index (BMI) values [36] (18.0 kg/m^2^ < lean ≤ 25.0 kg/m^2^, 25.0 kg/m^2^ < overweight ≤ 30.0 kg/m^2^, and obese > 30 kg/m^2^). T2D diagnosis was defined by the criteria [37] of Hemoglobin A1c (HbA1c) ≥ 6.5%.

### 2.3. Dietary Information

The seven-day dietary information of participants was collected by a self-report recording menu, ingredients, and quantity of every meal in the week. The energy (kcal) and amount (g, mg, and μg) of each nutrient were estimated according to the dietary records applied to the NutriSurvey-free software, version 2007 supplemented with the Indonesian food database (http://www.nutrisurvey.de/index.html: accessed 2 April 2020). In addition, T2D subjects were asked about ongoing dietary restriction therapy using a questionnaire.

### 2.4. Fecal Sample Collection and Transportation Process

Fecal sample collection and transportation process were modified from Kisuse et al. [3]. The subjects collected four parts of fresh feces voided onto a sheet (TYK stool collection sheet, Japan) using a small spatula equipped with a stool collection tube (76 mm × 20 mm, Sarstedt, Germany). The two of them were transferred into 2 mL of RNAlater (Invitrogen, Thermo Fisher Scientific, Vilnius, Lithuania) to preserve DNA for 16S rRNA amplicon sequencing. The other two were transferred into 2 mL methanol to inactivate enzymes and preserve metabolites for metabolome analysis. Immediately after collection, the feces in the solution were shaken several times to be suspended in tubes containing five zirconia balls, YTZ^®^-2.5 mm (Nikkato, Sakai, Japan). The collected samples were transferred to the laboratory in UGM within 24 h. After the arrival of samples, the feces in both solutions were briefly homogenized by vortexing for 30 s. Then, the samples for the microbiome analysis were stored at −20 °C and those for the metabolome analysis were stored at −80 °C. Immediately before transporting samples to Kyushu University in Japan, 1 mL of the feces were homogenized in methanol before being transferred into a 1.5 mL fresh microtube. The methanol was evaporated using a vacuum centrifugal evaporator (MV-100 Micro Vac; Tomy Medico, Tokyo, Japan) without heating. The methanol-treated fecal pellet and the feces in RNAlater were transferred to Kyushu University in Japan within 24 h by air transportation under temperature control (<8 °C). Then, the samples were kept at −80 °C for metabolome and −20 °C for the 16S rRNA gene test until the analyses.

### 2.5. 16S rRNA Gene Amplicon Sequencing and Sequence Data Process

Bacterial genomic DNA was extracted from fecal samples using the bead-beating method as described by Matsuki et al. [38] (see Supplementary Materials). High-throughput 16S rRNA gene sequence analysis was followed Kisuse et al. [3]. The V3-V4 region of the bacterial 16S rRNA gene was amplified from the fecal genomic DNA (1 ng) using TaKaRa Ex Taq^®^ HS (Takara Bio, Kusatsu, Japan) and universal primers: Bakt_341F (5-CGCTCTTCCGATCTCTGCCTACGGGNGGCWGCAG-3) and Bakt_805R (5-TGCTCTTCCGATCTGACGACTACHVGGGTATCTAATCC-3) [39]. The sequence data were processed using the UPARSE pipeline in USEARCH v9.2.64 software (http://drive5.com/usearch/download.html: accessed 19 August 2020) [40] (see details in Supplementary Materials). The taxonomy of OTUs was identified with cut-off values higher than 0.8 in SINTAX algorithm [41] with the reference sequence database of RDP training set v16 (https://sourceforge.net/projects/rdp-classifier/: accessed 19 August 2020). OTU table in Supplementary Table S4 shows the assigned taxonomy and rarefied counts of each OTU for each sample.

### 2.6. Statistical Analysis

Statistical analyses and graphics were made using RStudio software, version 1.0.153 (https://rstudio.com/: accessed 19 August 2020) with R software, version 3.5.1 (http://www.r-project.org: accessed 19 August 2020) and Stata/SE, version 12.0 (StataCorp LLC, College Station, TX, USA). To compare physiological indices, bacterial relative abundance, alpha-diversities, and the level of bacterial metabolites, a Wilcoxon rank-sum test was used to compare two groups. Pairwise Wilcoxon rank-sum with Bonferroni or Holm adjustment were used to compare more than two groups, except for the comparison of NOO among the different BMI groups in which a Welch’s *t*-test was used. Regression and correlation analysis of bacterial abundance and other indices were calculated by the lm function in R for normally distributed independent variables, or Spearman’s rank correlation in Stata for non-normally distributed variables. Validation of the established linear model was performed using the gvlma function in R. For the linear regression analysis, regression of microbiome or host physiological indices onto PCA ordination was performed with the ordisurf function from the vegan package in R.

### 2.7. Alpha-Diversity Analysis

As alpha-diversity indices, the number of observed OTUs (NOO) [42], Shannon Wiener index [43], and PD_whole_tree [44] were determined at a sequence depth of 9050 reads per sample with 10 random iterations using the alpha_rarefaction.py script in QIIME (http://qiime.org/scripts/alpha_rarefaction.html: accessed 19 August 2020).

### 2.8. Beta Diversity Analysis

PCA was performed based on the genus composition of the 75 samples using rda function in the R vegan package (https://cran.r-project.org/package=vegan: accessed 19 August 2020) and plotted by using ggplot function in ggplot2 package (https://cran.r-project.org/package=ggplot2: accessed 19 August 2020). Regressions of physical and microbiome indices to the PCA ordination were calculated using ordisurf function from the R vegan package and plotted by using ggplot function in the ggplot2 package.

### 2.9. Linear Discriminant Analysis Effect Size (LEfSe)

LEfSe was calculated using an online galaxy, version 1.0 (https://huttenhower.sph.harvard.edu/galaxy/: accessed 21 August 2020) [45]. Bacterial composition data of all subjects from phylum to OTU levels in which species are represented by OTU were subjected to linear discriminant analysis (LDA) using a one-against-all strategy. The taxa showing an LDA score higher than 3.0 at a *p* value less than 0.05 were selected as enriched taxa in each group.

### 2.10. Nuclear Magnetic Resonance (NMR) Metabolomics

Fecal samples were processed for quantitative NMR according to the method previously described [46]. Dried fecal pellets were thoroughly suspended in 700 μL of PBS buffer (100 mM, pH 7.4, in MagniSolv deuterated water; Merck, Darmstadt, Germany) containing 4 mM sodium 3-(trimethylsilyl) propionate-2,2,3,3-*d*_4_ (TSP-*d*_4_: Fujifilm Wako Pure Chemical, Osaka, Japan) as an internal standard by vortexing. Thereafter, the cell debris was removed by centrifugation at 9100× *g* for 10 min at 4 °C, twice, and 500 μL of supernatant was filled in a 5 -mm diameter NMR tube (Hilgenberg, Malsfeld, Germany). The remaining supernatant and fecal pellet in the tube were kept at −80 °C for the next bile acid quantification.

The 400 MHz ^1^H-NMR was quantitatively measured at 25 °C without spinning the tube on a JNM-ECZ400S (JEOL Ltd., Tokyo, Japan). The spectrum was obtained by a standard ^1^H-NMR pulse sequence with 90° pulse and 10 s delay time, while suppressing the water signal by using a pre-saturation method. The number of scans was 64. The obtained FID signal was subjected to Fourier transformation to yield the ^1^H NMR spectra, which was thereafter manually phased, baseline corrected, and integrated in JEOL Delta v5.3.1. The chemical shift and integration were referenced to TSP-*d*_4_ at 0.00 ppm and 9 protons, respectively. The concentrations of major SCFAs, namely acetate, propionate, butyrate, succinate, and lactate, were determined according to the integration of peaks at the corresponding chemical shifts.

### 2.11. Fecal Bile Acid Measurement

After the NMR analysis, the sample in the NMR tube was returned to the tube containing the fecal pellet and then dried using a Speedvac concentrator at a vacuum pressure rate of 50 torr/min without heating (Savant SPD1010; Thermo Scientific, Waltham, MA, USA). BAs were then extracted from the fecal pellet by ethanol containing an internal standard of 20 nM nor-deoxycholic acid (NDCA; Santa Cruz Biotechnology, Dallas, TX, USA) at 60 °C for 30 min and subsequently at 100 °C for 3 min. Thereafter, the ethanol extract was purified using an Oasis HLB cartridge column and then subjected to LC-MSMS analysis (LCMS-8050, Shimadzu, Kyoto, Japan). The methods in detail are described by Tanaka et al. [47].

## 3. Results

### 3.1. Physical Characteristics of Indonesian Subjects and Their Trends in Macronutrient Consumption

Physical and clinical characteristics of the subjects are shown in Table 1 and Supplementary Table S1. All subjects were grouped into two sets of dependent subjects by using characteristic criteria of HbA1c to classify the set of non-T2D and T2D groups, and BMI to classify the set of lean, overweight, and obese groups, respectively. Age significantly differed between the T2D and non-T2D groups, and it was adjusted in later statistical analyses to investigate the correlation between the microbiome and T2D. Overall, seven subjects in the T2D group were treated with the anti-diabetic drug, metformin.

To capture the trends in nutrient consumption of Indonesian subjects, statistical analyses using their seven-day dietary records were performed. As shown by the average of all subjects (Supplementary Table S2), the balance of macronutrients was in the range of WHO recommendations (carbohydrates, 55% to 75%; protein, 10% to 15%; fat, 15% to 30%; https://www.who.int/nutrition/topics/5_population_nutrient/en/: accessed 21 November 2020) although the fat consumption rate was close to its upper range at 30%. There was an obvious trend of increase in energy consumption from all three macronutrients from lean to obese groups. The fat consumption rate in the obese group was averaged to be 32.1% of the total energy corresponding to 670 kcal, which was significantly higher than 430 kcal in the lean group (*p* = 0.001, in pairwise Wilcoxon rank sum test with Bonferroni adjustment). On the other hand, between non-T2D and T2D groups, the carbohydrate consumption was significantly lower and the relative consumption of protein was significantly higher in the T2D group. Multiple linear regression analysis with the three macronutrient intakes showed that fat consumption was a major determinant of BMI in the non-T2D group (*p* = 0.042, beta = 0.43) (Supplementary Table S3). On the other hand, no correlation between the macronutrients and BMI was found in the multiple linear regression analysis in the T2D group. This appears to be because the majority of T2D patients in this study were on dietary restriction (Supplementary Table S1).

### 3.2. Gut Microbiome Variance of Indonesian Subjects Is Driven by Three Bacterial Genera

**Figure 1:**
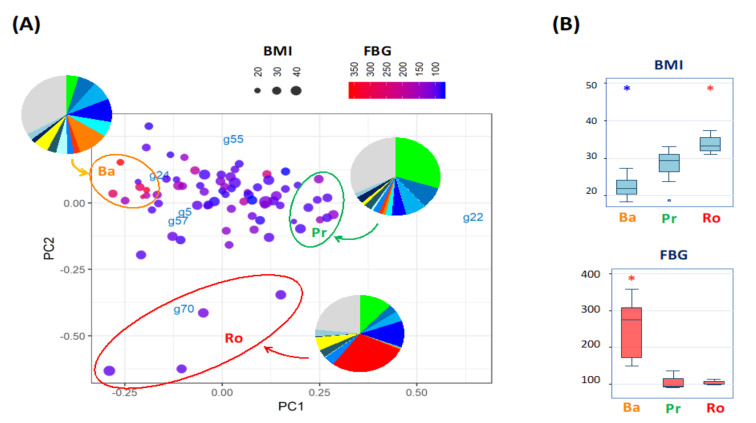
Fecal microbiome variation of the 75 Indonesian adults driven by three genera and their relation to obesity and diabetes. (**A**) Principal component analysis plot of the 75 microbiomes of Indonesian subjects. The sample ordination and genus loadings were calculated according to the genus composition of 75 stool samples. The BMI and FBG of each sample donor are represented by the dot size and color according to the indicated scales. The samples localized in each edge of these 75 samples ordination were selected and circled with the first two letters of the driving genera, namely Ba of *Bacteroides*, Pr of *Prevotella*, and Ro of *Romboutsia*. The genus composition was averaged within the circle and graphed in the pie charts. (**B**) Box plot of BMI and FBG in the three clusters. Red and blue asterisks represent statistically higher and lower than the other groups with *p* < 0.05 in the pairwise Wilcoxon rank-sum test with Bonferroni adjustment.

To determine the gut microbiome variance of Indonesian subjects, the bacterial taxonomic compositions of fecal samples from 75 Indonesian male adults, including obese and T2D subjects were analyzed by using amplicon sequencing of the 16S rRNA V3-V4 region. Subsequently, their microbiome variation were profiled by principal component analysis (PCA) based on the genus composition of each sample (Figure 1A). The biplot showed a triangular distribution of 75 samples, driven by three dominant genera: *Bacteroides* (g24), *Prevotella* (g22), and *Romboutsia* (g70). To examine the microbial community driven by the three genera, the samples placed at each edge of the triangle were grouped and their genus composition was averaged within each group (pie charts in Figure 1A). As a result, three types of bacterial community, each dominated by *Bacteroides*, *Prevotella*, and *Romboutsia*, were shown evidently. Furthermore, the *Romboutsia-*driven group was evidently characterized with high BMI, while subjects with high fasting blood glucose (FBG) levels were clustered in the *Bacteroides-*driven group (Figure 1B).

It is statistically confirmed that BMI, HbA1c, and FBG were regressed on the PCA ordination (Supplementary Figure S1). Both indicators of T2D increased toward the *Bacteroides* loading vector, while BMI increased toward the *Romboutsia* loading vector. Moreover, it was found that some indices reflecting the condition of gut microbiota were highly correlated with PCA ordination. The first is the number of observed OTUs (NOO), a known indicator of microbiome community richness, was high in the center of the PCA ordination and lower toward the edges of the triangle, particularly the direction of the *Romboutsia* loading vector (Supplementary Figure S1D). The level of fecal primary bile acids was increased in the *Romboutsia*-driven region, while it was mostly depleted in the *Bacteroides*-driven region (Supplementary Figure S1E). The level of succinate was also increased in the *Romboutsia*-driven region, while it was close to depleted in the belt spanning between *Bacteroides*- and *Prevotella*-driven edges (Supplementary Figure S1F). Primary bile acids were intermediate metabolites from conjugated bile acid and secondary bile acids. In addition, succinate is also known as an intermediate metabolite between propionate and butyrate. The microbial community appeared to lose the full metabolic functionality for SCFA and BAs in samples of the PC2-negative region with high concentrations of these metabolic intermediates.

### 3.3. Gut Microbiome Features of Obese or T2D Indonesian Subjects

**Figure 2:**
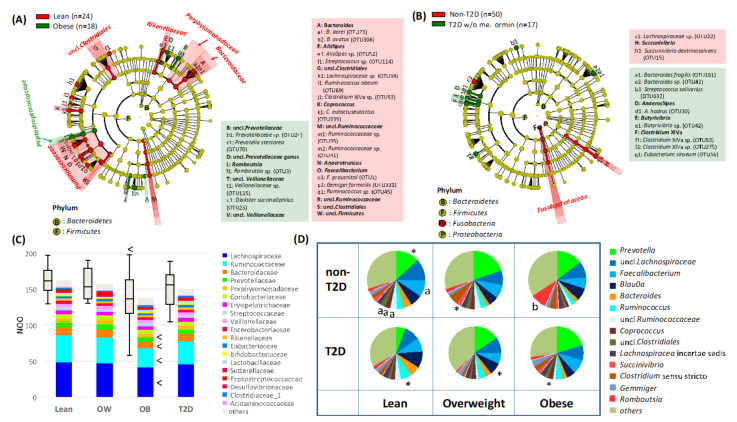
Fecal microbiome features associated with obesity and T2D. (**A**) LEfSe showing taxa distinguishing non-T2D obese subjects as compared to non-T2D lean subjects. The Lefse analysis was performed using bacterial composition data of fecal samples from phylum to OTU levels, in which species were represented by OTUs (Supplementary Table S4). The LDA scores were calculated by using the Wilcoxon rank-sum test and the taxonomic groups showing LDA scores higher than 3.0 with *p* < 0.05 were highlighted by the indicated color on the cladogram. (**B**) LEfSe showing taxa distinguishing T2D lean subjects as compared to non-T2D lean subjects. The LEfSe analysis were performed by the same methods as (**A**). (**C**) Comparison of the number of OTUs observed in each sample among non-T2D lean, non-T2D overweight, non-T2D obese, and T2D subjects. The number of observed OTUs (NOO) was estimated for each family and stacked in the bar graph accompanied with box plot showing the distribution of total NOO. Welch t-test was performed to examine the statistical difference as compared to the non-T2D lean group and total NOO and the families with statistically lower NOO were marked by less-than a sign. (**D**) Cross comparison of genus composition among lean, overweight, and obese subgroups, and between T2D and non-T2D subgroups. Asterisk represents results that are statistically higher in the indicated group in the comparison between non-T2D and T2D subgroups of the same BMI group (*p* < 0.05 in Wilcoxon rank-sum test). Letters, “a” and “b”, represent those statistically higher in the lean subgroup compared to the obese subgroup and vice versa within the non-T2D group (*p* < 0.05 in pairwise Wilcoxon rank-sum test with Bonferroni adjustment among lean, overweight, and obese subgroups).

To determine abnormalities in the gut microbiome of obese Indonesian subjects, bacterial compositions between non-T2D obese subjects (BMI > 30 kg/m^2^, HbA1c < 6.5%) and non-T2D lean subjects (BMI ≤ 25 kg/m^2^, HbA1c < 6.5%) were compared by using the LEfSe (Figure 2A). The results indicated that a broad range of taxonomic groups, including common gut microbiome families, Ruminococcaceae, Bacteroidaceae, and unclassified families of Clostridiales, declined in the obese group, while genus *Romboutsia* was significantly increased in the obese group. NOO was significantly lower in the obese group, in which the number of OTUs of commensal groups, such as Lachnospiraceae, Ruminococcaceae, and Bacteroidaceae, decreased significantly (Figure 2C). Namely, in the obese subjects, *Romboutsia* outgrew the commensal group, which is most likely dysbiosis.

On the other hand, the Lefse analysis to compare the gut microbiome differences between T2D and non-T2D groups showed that a narrow range of taxonomic groups, such as *Bacteroides fragilis* (OTU151) and four Lachnospiraceae species were altered in the T2D group (Figure 2B). Alpha-diversity indices did not significantly differ between these two groups (Figure 2B). These results indicated that Indonesian T2D subjects had a local alteration unlike dysbiosis in their gut microbiota.

To confirm the compositional change with obesity and diabetes, cross analysis over the different BMI and FBG subgroups were performed (Figure 2D). Among these subgroups, the *Romboutsia*-overgrown biome of the non-T2D obese subgroup, and the *Prevotella-*deprived biome of the T2D lean subgroup were remarkable, while the trend of dysbiosis, namely a decrease in Clostridiales, such as *Faecalibacterium* and *Coprococcus* with increasing obesity, was confirmed within the non-T2D group but not in the T2D group. The outgrowth of *Romboutsia* was also not observed in the T2D obese subgroup. Interestingly, the non-T2D obese subjects with a higher ratio of *Romboutsia* had a high concentration of succinate in their feces, which is known to be involved in the control of blood glucose levels and may suggest that *Romboutsia* in these obese subjects acts to control the host blood glucose level (Supplementary Figure S2).

### 3.4. Association of Gut Microbiome of Indonesian Subjects with Diets, Obesity, and T2D

**Figure 3:**
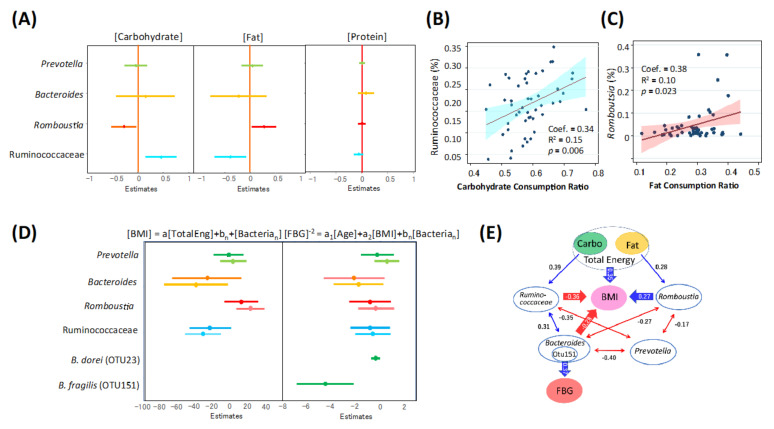
Association of intestinal bacteria with dietary macronutrients and host metabolic indices. (**A**) Single linear regression analysis was performed to estimate correlation of each bacteria group’s abundance to macronutrient consumption of the host using the dataset of non-T2D subjects. Estimates with CI95 were shown in the forest plot. (**B**,**C**) Scatter plot correlating with host carbohydrate consumption ratio and the abundance of Ruminococcaceae (**B**) and *Romboutsia* (**C**). CI95 range is colored. (**D**) Multiple linear regression analysis was performed to estimate the correlation of each bacterial abundance to BMI and FBG, respectively. Regression to BMI was estimated using the relative abundance of the four bacterial groups and host total energy consumption of non-T2D subjects. One subject (no. 303) was removed as an outlier to satisfy the assumption for linear regression. For regression to FBG, FBG values were converted to their inverse square values showing normal distribution and were then used as independent variables. The relative abundance of the four bacterial groups and host age and host BMI were used as dependent variables. The estimates with CI95 were shown (upper lines). Additionally, the relative abundance of the four bacteria and two *Bacteroides* species was solely used as the multiple regression analysis with host BMI and age and the estimates with CI95 were shown (lower lines for the upper four bacteria). (**E**) Path diagram showing correlations among the four driving bacterial groups, energies from diets, host BMI, and FBG. Red and blue arrows represent negative and positive correlations, respectively. The number besides the line shows the correlation coefficient between the connected two valuables.

To determine the association of the gut microbiome with diets and host metabolic status, four key taxa, namely the three driving genera *Prevotella*, *Bacteroides*, and *Romboutsia*, and one core family of Ruminococcaceae, were subjected to a series of linear regression analyses (Figure 3). First, the correlation of the four key taxa to each macronutrient intake ratio was calculated by using 75 individual datasets. Results are shown in Figure 3A–C, revealing that *Romboutsia* and Ruminococcaceae have opposite associations with fat and carbohydrate, respectively.

Subsequently, the correlation of the four key taxa with obesity and T2D was analyzed. To adjust for confounding effects among the host factors and inter-bacterial interactions, a multiple linear regression analysis was performed. For obesity, BMI as the dependent variable, total energy intake of the host, and abundances of the four key bacterial groups as the independent variables were used. To remove the confounding effect of T2D including drug and diet therapies, non-T2D subjects were used for this analysis. Normal distribution was confirmed with *p* > 0.05 in the skewness and kurtosis tests for the 50 samples of BMI values. The results of the initial trial using all samples did not satisfy the global validation of linear model assumptions, due to an outlier with unusual dietary records. Therefore, the second trial was performed by excluding the one outlier, which satisfied the linear model assumptions. Figure 3D shows the estimates of regression coefficients (95% CI) of each bacterial group to BMI, in which the upper and lower lines were estimated by applying these four bacteria data altogether or individually as dependent variables in the regression analysis. The results statistically support the model that *Bacteroides* and Ruminococcaceae negatively correlate with BMI, while *Romboutsia* positively correlates with BMI.

For the correlation with FBG as an index for T2D, age and BMI of each sample donor were included as dependent variables and the subjects administered metformin were excluded. Instead of FBG values, their inverse square values showing normal distribution were used as independent variables. The relative abundance data of the four key taxa were applied together or individually in the multiple regression analysis. However, none of the four bacterial groups showed statistically significant correlation, although the coefficient of *Bacteroides* was nearly significant (Figure 3D). Therefore, abundance data of each *Bacteroides* OTU were applied independently to the multiple regression model. *B. fragilis* (OTU151) showed a significantly negative correlation with the inverse square of FBG, whereas the dominant species of *Bacteroides*, (OTU23) also showed a significantly negative, but weak correlation.

A path diagram was created to show the linkage among diets, the microbiome, and host metabolism (Figure 3E). Ruminococcaceae was positively correlated with carbohydrate intake ratio but negatively with BMI, whereas *Romboutsia* was positively correlated with fat intake ratios and BMI. *Bacteroides* tended to be co-abundant with Ruminococcaceae and negatively correlated with BMI. *Bacteroides* also showed a marginally positive correlation with FBG, wherein subdominant *B. fragilis* showed a strong positive correlation with FBG. *Prevotella*, which was the most dominant genus in Indonesian subjects, competed with the other dominant groups, notably *Bacteroides*, but did not correlate directly with the indices of diet, obesity, and T2D.

### 3.5. Potential Microbiome Markers for Fat-Driven Obesity in Indonesian Subjects

To cover the correlation of microbiome to diet, obesity, and diabetes in more detail, all families, genera, and OTUs were applied to the same regression model (see the OTU table in Supplementary Table S4 and the results in Supplementary Tables S5–S9). Many taxa were correlated particularly with carbohydrate and fat intake ratios and obesity. In obesity, two genera, namely *Oscillibacter* and *Coprococcus*, showed an apparent negative correlation with BMI, in addition to *Romboutsia*’s positive correlation with BMI, while *Bacteroides* and Ruminococcaceae were negatively correlated with BMI (Supplementary Figure S3). Moreover, at the OTU levels, OTU36 and OTU89, which are closely related to *Coprococcus* sp. and *Oscillibacter valericigenes*, respectively, were positively correlated with carbohydrate intake ratio, negatively with fat intake ratio, and BMI negatively (Supplementary Tables S5 and S6, Supplementary Figure S4). Multiple linear regression using the abundance of these two OTUs as independent variables explained the variance of non-T2D subjects at 31.0% for fat intake ratio, 32.0% for carbohydrate intake ratio, and 38.0% for BMI. This suggests that these two OTUs are microbiome markers for fat-driven obesity in Indonesia.

### 3.6. Alteration of Bile Acid Metabolism in Microbiota of Obese and T2D Indonesian Subjects

**Figure 4:**
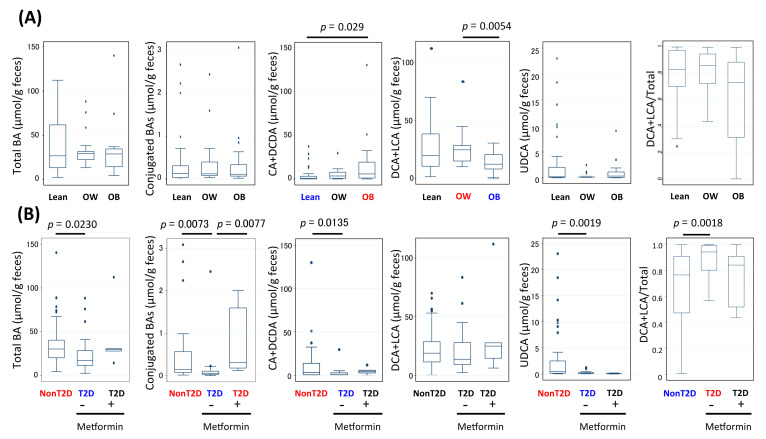
Comparison of fecal BA concentrations between different BMI groups (**A**) and among the non-T2D group and metformin-treated and non-treated T2D groups (**B**). The distribution of each BA group concentration (μmol/g dry feces) was graphed in box plots. The statistical difference between groups was calculated by the pairwise Wilcoxon rank-sum with Holm adjustment and *p* value lower than 0.05 was denoted.

First, the amount of each BA group was compared among the different BMI groups (Figure 4A, Supplementary Table S10). To coincide with the previous analysis in Supplementary Figure S1, the level of the primary BA group (cholic acid + chenodeoxycholic acid, CA+CDCA) was increased in the obese group compared with the lean group, while the 7α-dehydroxylated BA group (deoxycholic acid + lithocholic acid, DCA + LCA) was significantly lower in the obese group than in the overweight group. Additionally, the relative ratio of the 7α-dehydroxylated group to total BA (7dOH ratio: DCA + LCA/Total BA) was estimated in order to represent the total BA conversion rate to the main end product, and was found to be reduced in the obese group, although it was not significant due to high variance. On the other hand, in the T2D group, the 7dOH ratio was mostly close to one and significantly higher than that in the non-T2D group, while conjugated BAs, primary BAs, and ursodeoxycholic acid (UDCA) were mostly depleted (Figure 4B, Supplementary Table S11). Interestingly, the conjugated BA level was recovered in the T2D patients with metformin administration, while the primary BA level and UDCA level were not recovered. The correlation of each BA level with FBG level was further analyzed using Spearman correlation analysis (Supplementary Table S12). Overall, two glycine-conjugated BAs, namely glycolithocholic acid (GLCA) and glycoursodeoxycholic acid (GUDCA), and two taurine conjugated BAs, taurolithochoic acid (TLCA) and tauroursodeoxycholic acid (TUDCA), showed negative correlations with FBG, while UDCA showed a stronger negative correlation.

**Figure 5:**
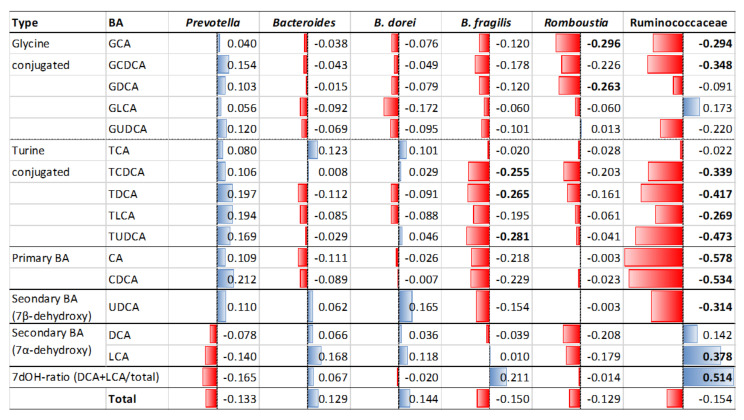
Correlation of the abundance of key taxa with the concentration of bile acid molecules in feces. Spearman correlation between relative abundance of these key taxa and concentration of each bile acid molecule was investigated using fecal samples of 71 Indonesian subjects. Spearman’s rho value was shown. Bold letters represent statistically significance (*p* < 0.05).

**Figure 6:**
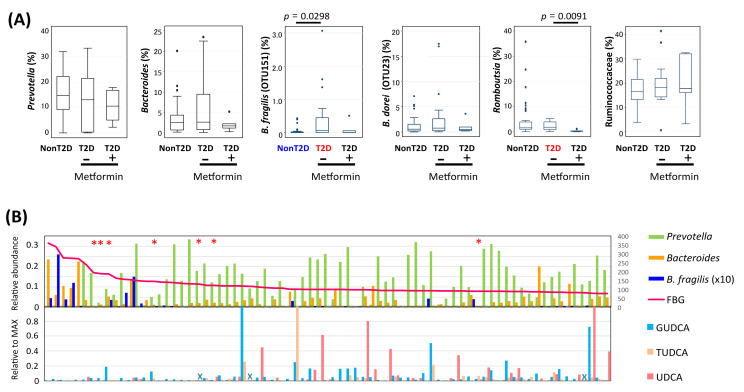
Distribution of the key bacteria and anti-diabetic UDCAs among Indonesian subjects. (**A**) Comparison of abundance of the key taxa among non-T2D, metformin-treated T2D, and non-treated T2D groups. (**B**) Relative abundance of genera *Prevotella*, *Bacteroides* and *B. fragilis* (upper graph) and GUDCA, TUDCA, and UDCA (lower graph) in 75 Indonesian subjects ordered by the FBG level. Red line indicates FBG. Red asterisks above the graph indicate subjects administered metformin. Blue crosses in the bottom graph indicate the samples lacking in the bile acid data.

Subsequently, the correlation of the key bacteria groups was calculated with the abundance of each BA molecule in feces (Figure 5). *Prevotella* showed a unique correlation profile that was positive for all BAs, except for 7α-dehydroxylated BAs, although they were not statistically significant. *Bacteroides* did not show any significant correlation to these BAs and *B. dorei* (OTU23) did not either, whereas *B. fragilis* (OTU151) showed a significant negative correlation with some conjugated BAs including TUDCA, which is known to have a function to control blood glucose levels as an antagonist of FXR. Ruminococcaceae showed significant correlations with many BAs, such as a strong negative correlation with primary BAs, moderate negative correlation with UDCA, and some conjugated bile acids. In addition, Ruminococcaceae showed a strong positive correlation with the 7dOH ratio, suggesting the presence of Ruminococcaceae species, such as OTU64 and OTU41, which are strongly involved in 7α-dehydroxylation (Supplementary Figure S5A,B). However, the abundance of Ruminococcaceae did not differ between the T2D and non-T2D groups (Figure 6A). On the other hand, it was found that the abundance of *Bacteroides*, notably *B. fragilis*, was higher in the T2D group, but decreased to the basal level in the patients treated with metformin, as opposed to the conjugated BA level. *B. fragilis* promotes T2D through the reduction in conjugated UDCAs with antagonistic activity against FXR, while metformin cures T2D by decreasing *B.* fragilis [48]. In Indonesian subjects, the conjugated BAs were mostly depleted when carrying a high number of OTU151 (Supplementary Figure S5C). However, note that deconjugated UDCA showed higher negative correlation to FBG (Supplementary Table S12), although not *B. fragilis* but Ruminococcaceae and the two OTUs, OTU64 and OTU41, showed significant negative correlation with UDCA and positive correlation with 7dOH-ratio (Figure 5 and Supplementary Figure S5A,B,D,E). It appears that the increase in Ruminococcaceae with strong 7α-dehydroxylation activity may outcompete the 7-epimerization of CDCA to UDCA catalyzed by some other commensal bacteria, such as *Fusicatenibacter saccharivorans* (OTU11), that showed a positive correlation with the fecal UDCA level (Supplementary Figure S5F).

Finally, the abundances of the three key bacteria and three anti-diabetic UDCAs in the order of FBG were profiled (Figure 6B). This clearly indicates that the patients with high FBG levels and without metformin administration were highly colonized by *B. fragilis*, and lacked *Prevotella* and both conjugated and unconjugated UDCA.

## 4. Discussion

Crosstalk between human gut microbiota, obesity, and diabetes has been studied, but it is still not much in developing Asian countries, tending to increase metabolic disease populations in reflection of changes in dietary environment. In this study, a cross-sectional study of Indonesian subjects was conducted to investigate microbiome and metabolome features associated with obesity and T2D, as well as their dietary habits and medical records. As a result, variations in fecal microbiome and metabolome found in the 75 subjects reflected the metabolic and dietary indices of the hosts.

The gut microbiome of the obese group was characterized by a dysbiosis-like microbiota community, in which *Romboutsia* abnormally increased in correlation with fat intake. *Romboutsia*, which is a member of the family Peptostreptococcaceae, is an obesity-related genus that positively correlates with lipid profiles and lipogenesis in the liver [49], as well as BMI [50]. Instead of *Romboutsia* overgrowth, potentially beneficial commensal bacteria were largely decreased in the obese group, notably butyrate-producing bacteria, including *Faecalibacterium*, *Roseburia*, *Coprococcus*, and *Oscillibacter*. Moreover, this dysbiosis-like status was reflected by the dysfunction of bile acid metabolism, as discussed later in this discussion section.

Although obesity is a risk factor for T2D, lean T2D is also highly prevalent in Asia [22,23]. In addition, the gut microbiota was characterized distinctively between obese and T2D subjects in this study, including a large portion of lean T2D subjects. The gut microbiota of T2D subjects with high FBG was characterized by *Bacteroides* overrepresenting in place of *Prevotella*, which is usually dominant in healthy Indonesian people [1]. Notably, the *Prevotella* level was significantly decreased in the lean T2D subjects (Figure 2D). Several studies have indicated that *Bacteroides* shows an antagonistic correlation with *Prevotella*, as observed as enterotypes [1,2,3,28,29,51,52]. Notably, a recent study has indicated that *Bacteroides*’s enterotype is associated with a high risk of T2D due to increased levels of lipopolysaccharide in blood, causing decreased insulin sensitivity, while *Prevotella* is antagonistic against the formation and function of the *Bacteroides* enterotype [52]. It is known that *Prevotella* strongly depends on carbohydrates in diet [51,53] and is a potent propionate producer with indigestible carbohydrate fermentation [54]. Propionate has been shown to trigger the secretion of the gut peptides glucagon-like peptide-1 (GLP-1) and peptide YY (PYY), which are involved in the regulation of appetite, glucose metabolism, and reducing inflammation [55]. Furthermore, *Prevotella* occasionally produces succinate as an intermediate fermentation product, which is known to improve glucose homeostasis via intestinal gluconeogenesis [56], although no positive correlation between *Prevotella* and succinate was observed in this study. Interestingly, in this study, high levels of succinate were instead found in the feces of non-T2D obese subjects in association with an increase in the *Romboutsia* population. However, these are a line of studies showing non-beneficial aspects of bacteria-derived succinate, overrepresented as a result of dysbiosis. Notably, a recent human study showed that blood succinate level increases in association with FBG and certain groups of gut bacteria including *Prevotella* [57]. There are controversies over whether *Prevotella* and succinate benefit human health [13]. Further studies on Indonesian obesity and T2D, each showing different aspects in the gut microbiome, may allow to understand the link between these major bacteria and metabolites in the intestine of humans with metabolic diseases.

**Figure 7:**
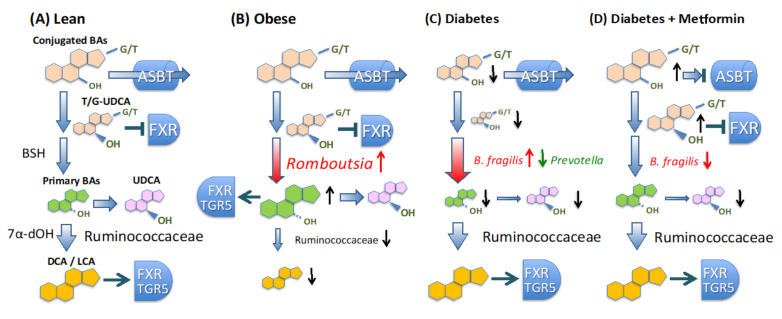
Hypothesized model of the key taxa-related BA metabolism in Indonesian gut linking to obesity and T2D. (**A**) In healthy lean subjects, conjugated BAs, secreted into the upper intestine are reabsorbed into the liver via apical sodium bile salt transporter (ASBT). Unabsorbed BAs are deconjugated by bacterial bile salt hydroxylase (BSH). Non-digested conjugated BAs, particularly TUDCA and GUDCA, contribute to glucose homeostasis through the antagonistic inhibition of FXR signaling. In the lower intestine, the deconjugated BAs are further metabolized by bacterial 7α-dehydroxylase (7α-dOH) or 7β-hydroxysteroid dehydrogenase to form 7α-dehydroxylated BAs, namely DCA, LCA, and UDCA, respectively. The highly diversified commensal taxon, Ruminococcaceae, is mainly involved in the 7α-dehydroxylation. The primary and secondary BAs act agonistic with different affinity to TGR5 and FXR, which coordinate metabolic homeostasis. (**B**) In the obese subjects, fecal primary BA level was increased with the increase in *Romboutsia* and decrease in Ruminococcaceae. (**C**) In the T2D subjects, TUDCA and GUDCA was decreased with the increase in *B. fragilis* equipped with strong BSH activity. The lack of antagonistic activity of TUDCA and GUDCA to FXR impairs glucose homeostasis. UDCA showing anti-diabetic aspect was also decreased with increase in *Ruminococcaceae.* (**D**) Metformin elevates the concentration of total BAs by inhibiting ASBT and inhibits the growth of *B. fragilis*, which eventually improve glucose homeostasis.

The fecal BA profile of Indonesian patients significantly reflected the gut microbiome status under metabolic diseases, as summarized in Figure 7. Generally, BAs synthesized in conjugated form in the liver are secreted into the duodenum via the gallbladder. Thereafter, they are deconjugated by bacterial bile salt hydroxylase (BSH) and further metabolized by bacterial 7α-dehydroxylase or 7β-hydroxysteroid dehydrogenase to form secondary BAs. In obese subjects, the primary BA level was remarkably increased with the increase in *Romboutsia*, suggesting impairment of BA metabolism in the intestinal microbiome. As mentioned previously, the *Romboutsia*-enriched microbiome had dysbiosis-like features lacking in the commensal group. Notably, a concomitant decrease in Ruminococcaceae, including some OTUs apparently involved in 7α-dehydroxylation (Supplementary Figure S5), appears to cause dysfunction of 7α-dehydroxylation. Ruminococcaceae was positively correlated with carbohydrate consumption, while *Romboutsia* did with fat consumption, as shown in Figure 3E. It appears that Ruminococcaceae basically constitutes the core microbiome of Indonesian people depending on a high-carbohydrate diet, as well as *Prevotella.* Dehydroxylated BAs tend to have higher activity for both TGR5 and FXR activations [58], the impairment of BA metabolism in obese subjects appeared to have an adverse impact on metabolic homeostasis. Of note, a recent study has demonstrated that oral gavage of *Parabacteroides distasonis* alleviates obesity and metabolic dysfunction in mice via the production of succinate and secondary bile acids, suggesting that these microbiome metabolites are involved in host metabolic homeostasis [59], as well as these findings.

In T2D subjects, depletion of conjugated BAs and UDCA was obvious where UDCA was statistically more correlated with FBG. UDCA, and TUDCA indeed improve glucose metabolism [60,61,62]. As mentioned in the results section, 7α-dehydroxylation by Ruminococcaceae appears to compete with 7-epimerization of CDCA, resulting in UDCA. On the other hand, the depletion of conjugated BAs, including TUDCA, can be explained by over-representation of *B. fragilis* as elucidated by a previous study [48]. This study suggests that *B. fragilis* is involved in T2D through its BSH function, which causes the loss of conjugated BAs, notably GUDCA and TUDCA, functioning as an FXR antagonist and improving glucose homeostasis. Metformin has been reported to inhibit the growth of *B. fragilis* due to suppression of folate metabolism required for methionine biosynthesis [48]. Indeed, *B. fragilis* was strongly reduced in metformin-administered patients. Metformin is also known to reduce proximal bile acid resorption, and it enhances the interaction of BAs with TGR5 in the distal gut, leading to an increase in GLP-1 secretion and a reduction in blood glucose [63]. Some reports have shown that metformin administration increases fecal BA levels, which coincides with the observations in subjects [64,65] (Figure 4B). However, it should be noted that a number of gut bacterial species other than *B. fragilis* have BSH activity [66], suggesting a further underlying mode of action involved in the microbiome-diabetes axis in Indonesian people. It should also be noted that the subjects treated with metformin mostly did not recover from T2D, but recovered the level of conjugated BAs without UDCA. Recovery of UDCA levels in addition to conjugated BAs might be required for the recovery of diabetes and might be a target for the therapy following metformin.

There are limitations to this study noted as follows. The sample size was not as large enough to satisfy adequate statistical power, suggesting that more samples would be required to confirm the results of this study. Information on disease and treatment history were not captured precisely and not allowed to address the link between the microbiome, disease treatment, and disease progress. Moreover, the sampling city was limited to Yogyakarta, suggesting that studies in different cities are required to capture the status of the entire Indonesian population.

## 5. Conclusions

This study indicates two types of gut microbiota, each of which is differently associated with obesity and T2D. High-fat diet-driven Indonesian obesity is associated with *Romboutsia*-driven gut microbiome dysbiosis with the loss of intestinal secondary BAs in association with a decrease in commensal Ruminococcaceae. T2D in the Indonesian subjects is associated with an increase in *Bacteroides* with the loss of conjugated BAs known to have anti-diabetic activity, and this alteration is reversed in patients receiving metformin treatment. Taken together, the altered fecal bile acid profiles in Indonesian male subjects represent gut microbiome status linking host metabolic disorder. The precise mechanism of the microbiome’s interplay with food and drug components warrants further study.

## Figures and Tables

**Table 1:** Demographic and clinical characteristics of 75 Indonesian subjects in this study.

	Non-T2D			T2D			*p* Value ^1^	*p* Value ^2^
Category:	Lean	Overweight	Obese	Lean	Overweight	Obese		
No.	25	7	18	11	11	3		
Gender	Male	Male	Male	Male	Male	Male		
Age (years)	47.4 ± 8.0	42.6 ± 2.6	44.2 ± 6.2	52.4 ± 7.7	52.5 ± 5.8	46.0 ± 7.8	0.0015	0.0824
Body height (cm)	166.2 ± 6.1	171.0 ± 5.4	167.0 ± 4.2	170.2 ± 5.2	163.1 ± 4.0	165.3 ± 5.5	0.4100	0.4739
Body weight (kg)	63.2 ± 7.6	83.7 ± 5.6	92.5 ± 10.9	63.9 ± 8.1	71.5 ± 3.5	104.0 ± 31.4	0.1500	2.31 × 10^−12^
BMI (kg/m^2^)	22.9 ± 1.7	28.6 ± 1.6	33.1 ± 2.9	22.0 ± 2.0	26.9 ± 1.5	37.9 ± 10.2	0.2700	1.89 × 10^−14^
Anti-diabetic drugs (no.)	0	0	0	3	4	0		
HbA1c (%)	5.7 ± 0.3	5.3 ± 0.3	5.7 ± 0.3	9.6 ± 2.7	8.7 ± 1.3	7.3 ± 1.0	2.1 × 10^−12^	0.2961
Fasting blood glucose (mg/dL)	93.1 ± 11.9	89.0 ± 3.7	96.6 ± 10.9	221.5 ± 84.3	161.9 ± 53.8	138.0 ± 22.3	4.3 × 10^−12^	0.4084

^1^ The statistical significance between non-T2D and T2D groups was assessed by Wilcoxon rank-sum test. ^2^ The statistical significance between lean, overweight, and obese groups was assessed by Kruskal Wallis and subsequently pairwise Wilcoxon rank-sum with Bonferroni adjustment.

## Data Availability

Raw sequence data from this study were deposited in the DNA Data Bank of Japan (DDBJ; https://www.ddbj.nig.ac.jp/index-e.html: accessed April 2nd, 2020). The DDBJ sequence read archive was DRA009596 under BioProject no. PRJDB9293, containing the accession links of fecal sampling data under Biosample from SAMD00204586 to SAMD00204659.

## Supplementary Materials

The following are available online at https://www.mdpi.com/article/10.3390/microorganisms9050897/s1, Figure S1. Regression of physical and gut microbiome indices on4 the PCA ordination, Figure S2. Fecal succinate concentration and its relation to BMI,11 FBG, and Romboutsia abundance, Figure S3. Correlation of genera and family with BMI, Figure S4. Two OTUs as potential microbiome markers for fat-21 driven obesity of Indonesian people, Figure S5. Specific correlation between OTU and BA, Table S1. Physical and clinical characteristics of 75 Indonesian subjects, Table S2. Average macro nutrient consumption of Indonesian subjects a day, Table S3. Multiple linear regression of three macronutrients intake for BMI, Table S4. OTU table of 16S rRNA genes with taxonomy amplified from fecal sampples of Indonesian subjects (rarified at 10,000 reads per sample), Table S5. Correlation of bacteria families, genera, and OTUs to carbohydrate intake ratio (*p* < 0.05 in linear regression analysis), Table S6. Correlation of bacteria families, genera, and OTUs to fat intake ratio (*p* < 0.05 in linear regression analysis), Table S7. Correlation of bacteria families, genera, and OTUs to protein intake ratio (*p* < 0.05 in linear regression analysis), Table S8. Bacterial families, genera, and OTUs significantly correlated with BMI (*p* < 0.05 in linear regression analysis adjusted by total energy intake), Table S9. Bacterial families, genera, and OTUs significantly correlated with FBG (*p* < 0.05 in linear regression analysis using inverse squares of FBG as independent variable and adjusted by age and BMI), Table S10. Level of fecal bile acids in lean, overweight, and obese subjects, Table S11. Level of fecal bile acids in non-T2D subjects and T2D subjects with and without metformin administration, Table S12. Spearman correlation of each fecal bile acid level to FBG.
