# Supplementary material for: Gut Microbiome of Indonesian Adults Associated with Obesity and Type 2 Diabetes: A Cross-Sectional Study in an Asian City, Yogyakarta
Source: Microorganisms. 2021 Apr 22;9(5):897. doi: 10.3390/microorganisms9050897 (PMC8147061; doi:10.3390/microorganisms9050897)
Supplement: Supplementary file 1 [file microorganisms-09-00897-s001.zip › Indonesia_SupFig_Microorganisms.pdf]

Supplementary Figure legends

Supplementary Figure S1. Regression of physical and gut microbiome indices on the PCA ordination The PCA ordination in Fig 1(A) was regressed by BMI (A), HbA1c (B), FBG (C) of sample donors, and the number of observed OTUs (NOO) (D), primary bile acid level (PriBA) (E) and succinate level (F) of fecal samples. The regression splines were calculated by the ordisurf function as well as the values of *p* and deviance.

Supplementary Figure S2. Fecal succinate concentration and its relation to BMI, FBG, and *Romboutsia* abundance (A) Box plot for succinate concentration (mM/g dry feces) in feces of each group. (B) Two-dimensional plot for host BMI and fecal succinate. (C) Two-dimensional plot for host FBG and fecal succinate. (D) Two-dimensional plot for relative abundance of *Romboutsia* and succinate in feces.

Supplementary Figure S3. Correlation of genera and family with BMI Relative abundances of these genera and families were subjected to Spearman's rank correlation.

Supplementary Figure S4. Two OTUs as potential microbiome markers for fat-driven obesity of Indonesian people The relative abundance of these OTUs were subjected to the single linear regression analysis for the host carbohydrate and fat intake ratio and the multiple regression analysis for BMI with total energy intake. The correlation coefficient (β), probability (*p*) and R^2 were calculated and shown.

Supplementary Figure S5. Specific correlation between OTU and BA

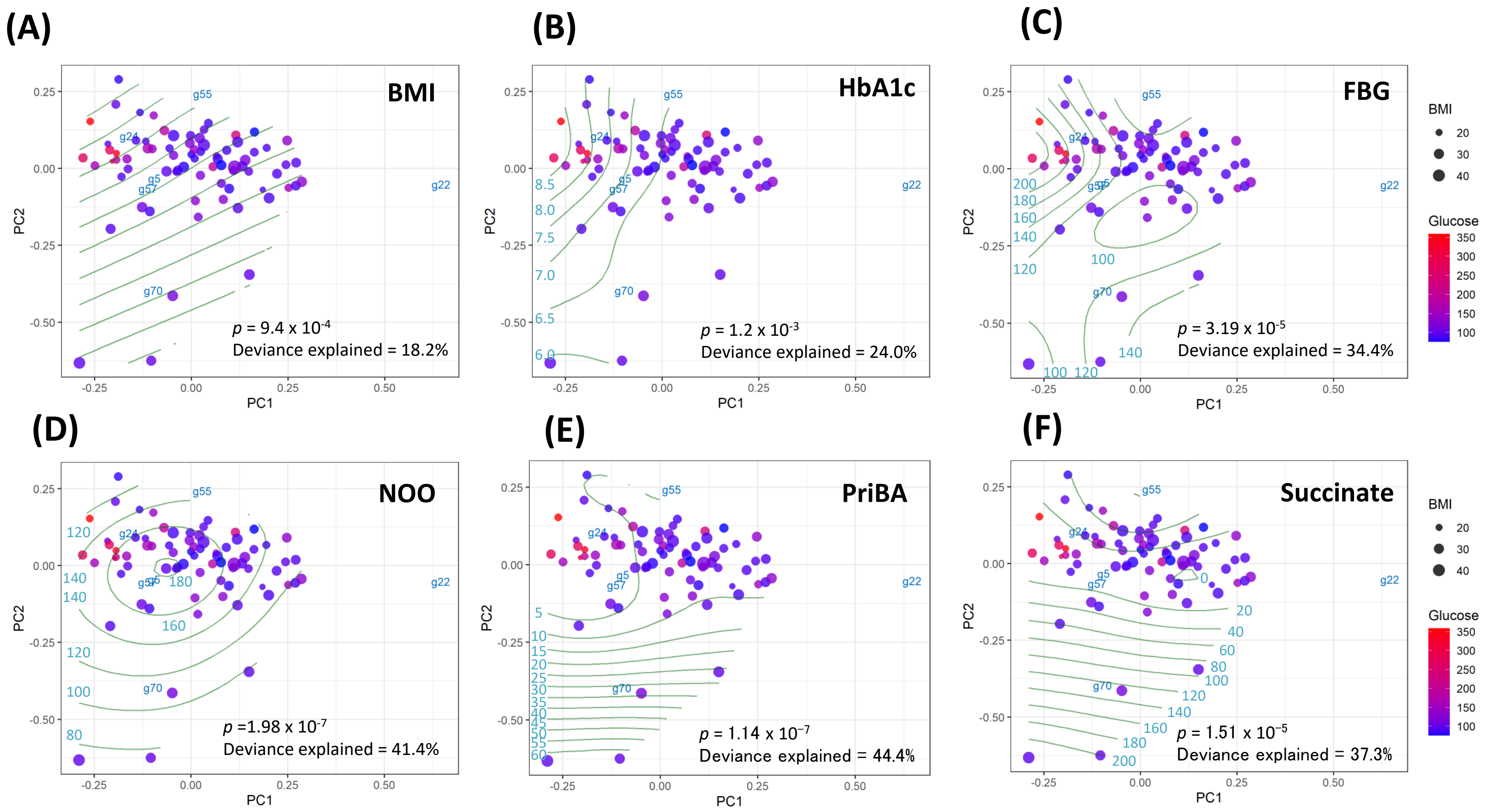

Supplementary Figure S1.

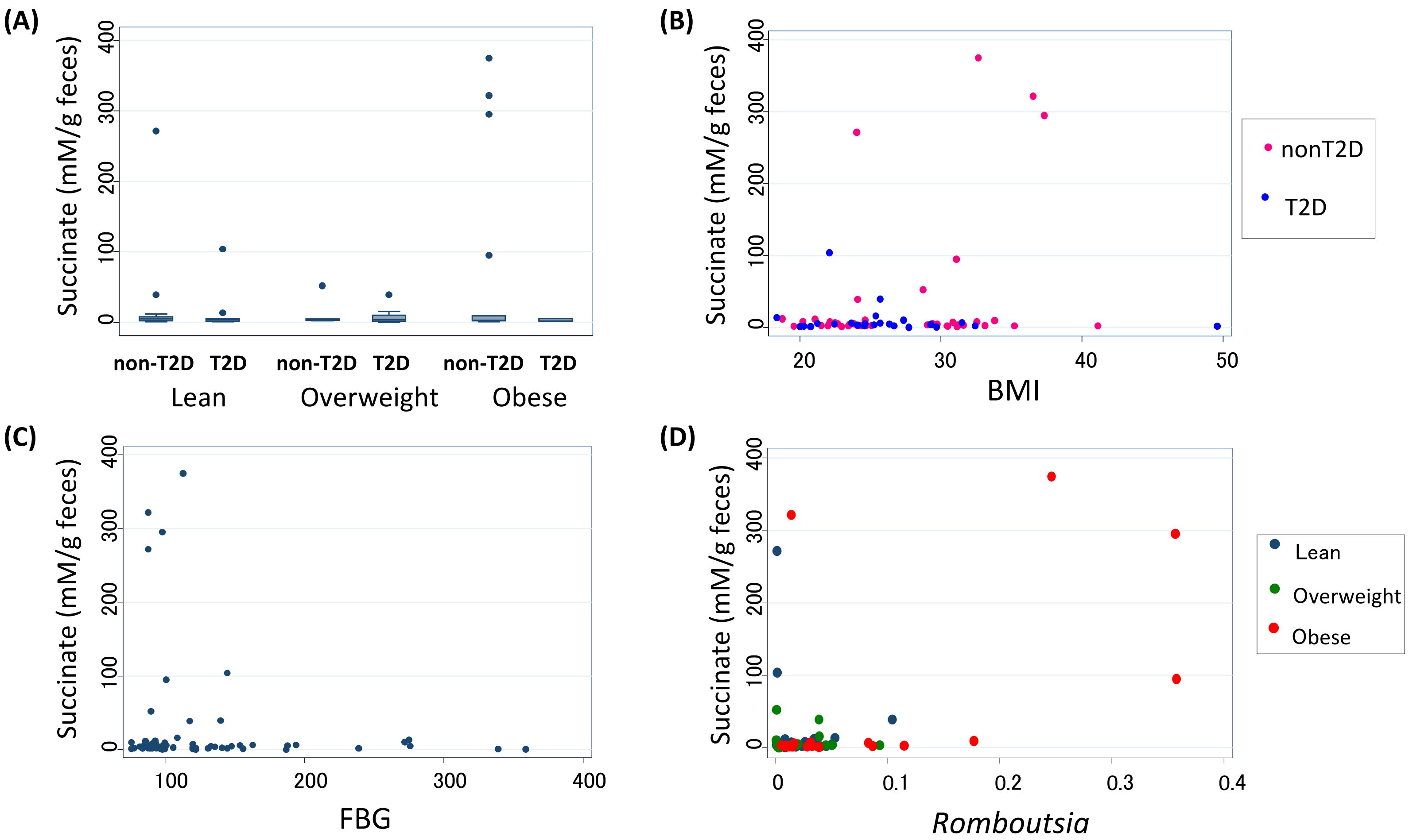

Supplementary Figure S2.

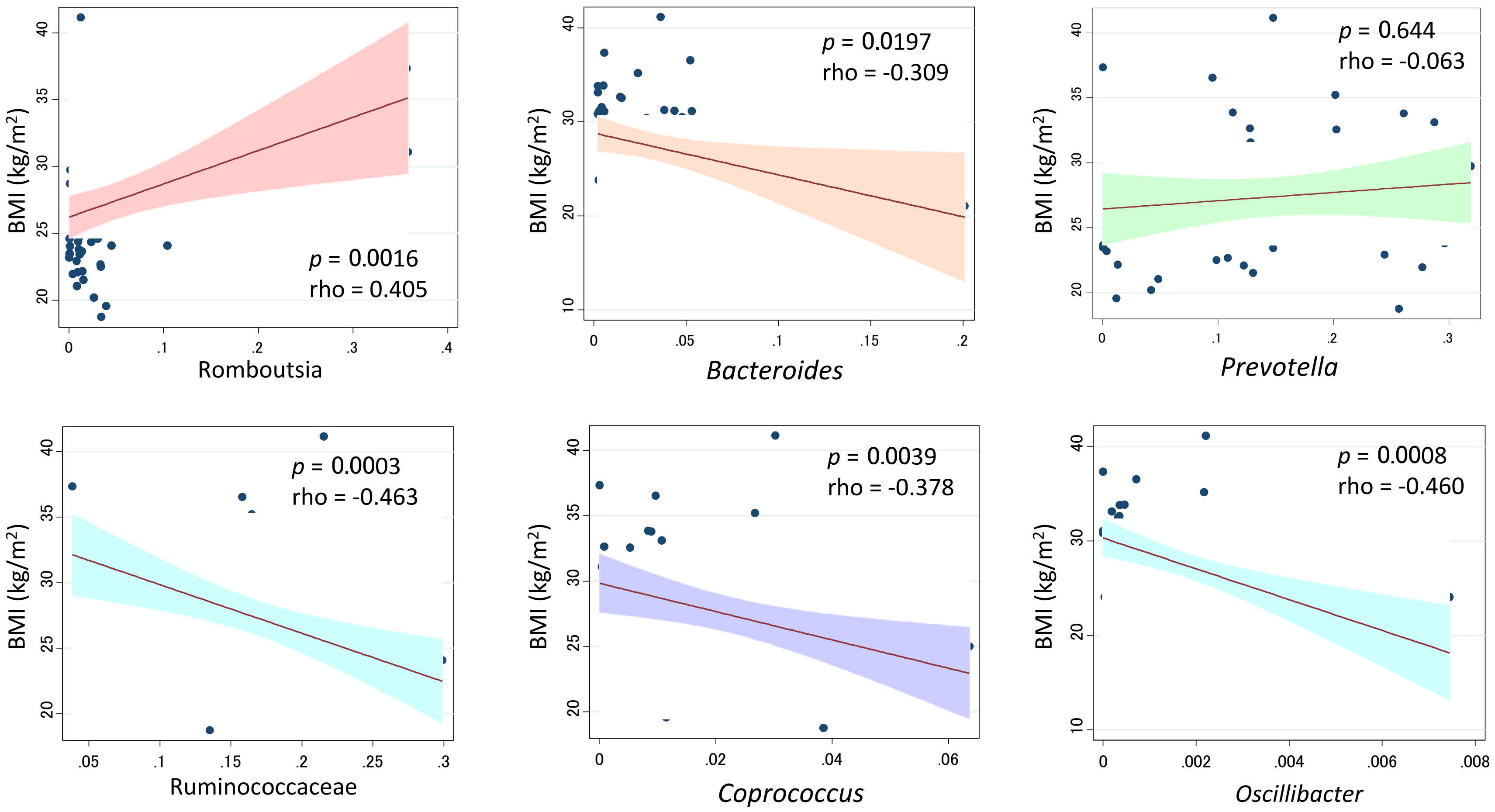

Supplementary Figure S3.

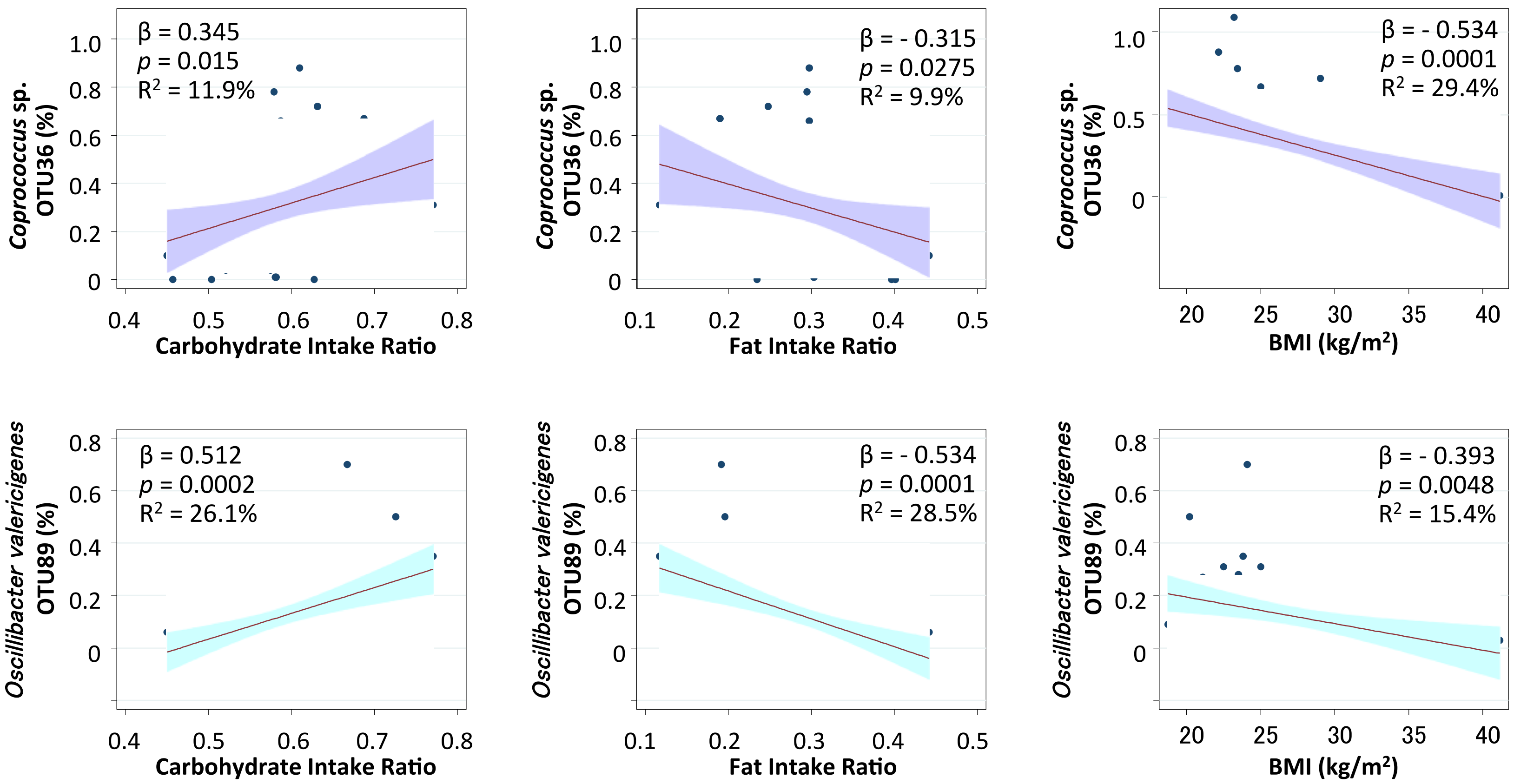

Supplementary Figure S4.

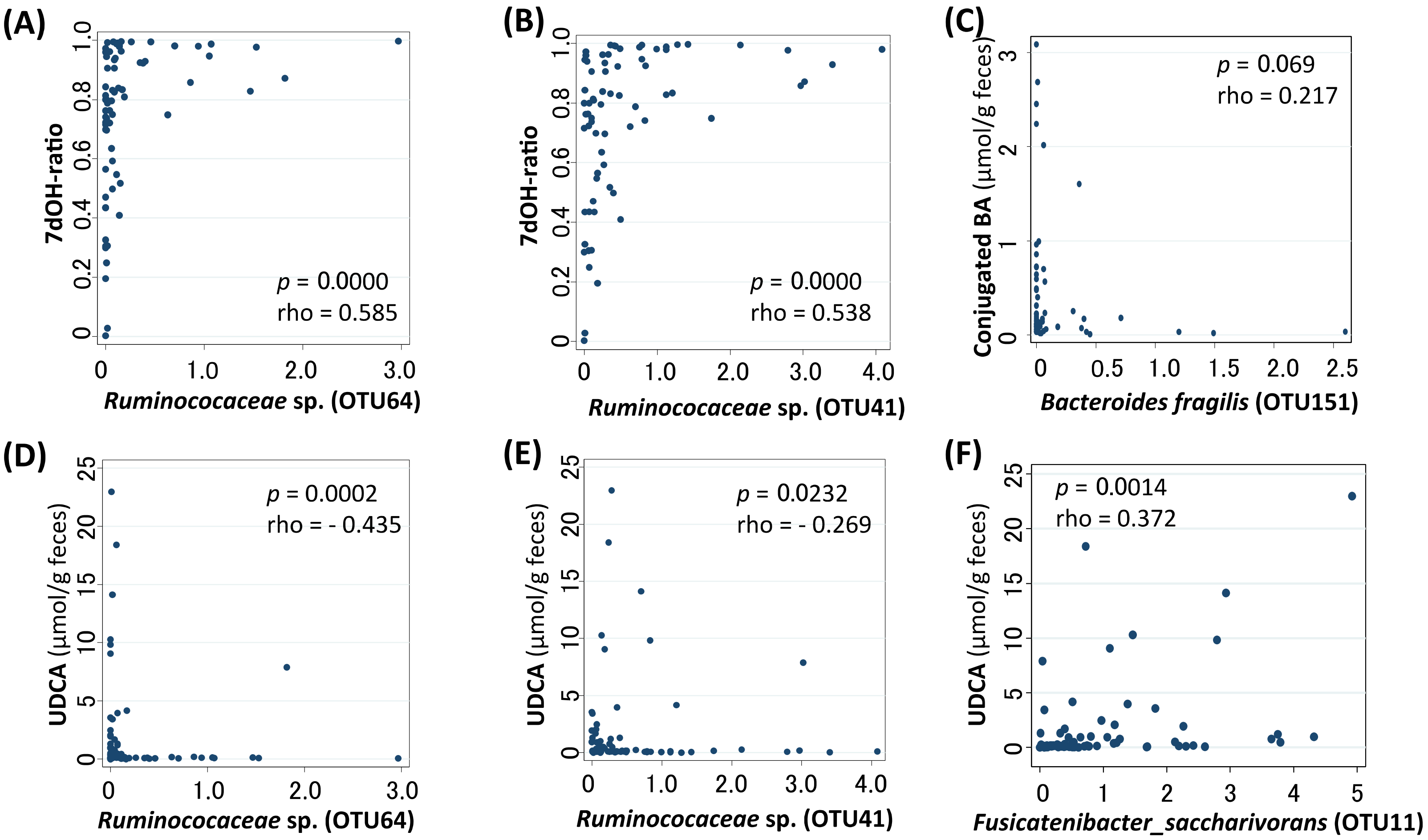

Supplementary Figure S5.
