# Supplementary material for: Gut Microbiome of Indonesian Adults Associated with Obesity and Type 2 Diabetes: A Cross-Sectional Study in an Asian City, Yogyakarta
Source: Microorganisms. 2021 Apr 22;9(5):897. doi: 10.3390/microorganisms9050897 (PMC8147061; doi:10.3390/microorganisms9050897)
Supplement: Supplementary file 1 [file microorganisms-09-00897-s001.zip › Supplementary Methods_Microorganisms.pdf]

Gut Microbiome of Indonesian Adults Associated with Obesity and Type 2 Diabetes: A Cross-Sectional Study in an Asian City, Yogyakarta

Phatthanaphong Therdtatha, Yayi Song, Masaru Tanaka, Mariyatun Mariyatun, Misaroh Almunifah, Nancy Eka Putri Manurung, Siska Indriarsih, Yi Lu, Koji Nagata, Katsuya Fukami, Tetsuo Ikeda, Yuan-Kun Lee, Endang Sutriswati Rahayu, and Jiro Nakayama

Supplementary Methods

Subject screening

In this study, subject screening was done based on the inclusion and exclusion criteria of the AMP phase IV shown as below.

1. Subjects must not have the following conditions:
    - Immunodeficiency
    - Recovery after surgical operation
    - Systemic infections
    - Systemic inflammatory disorders
    - Autoimmune disorders
    - Malignant cancers
    - Malnutrition
    - Renal insufficiency/failure
    - Acute gastrointestinal diseases, for example gastroenteritis, diarrhea, and etc.

2. Subject must not be received immune-alteration medications, such as immunosuppressive drugs, anti-inflammatory drugs, and etc.
3. Subjects must not be received the following medications within six months prior to the specimen collection:
    - Systemic antimicrobials (antibiotics, antifungals, antivirals or antiparasitics) via either intravenous (IV) or intramuscular (IM) routes
    - Systemic corticosteroid
    - Cytokines
    - Methotrexate or immunosuppressive cytotoxic agents
4. Subjects must not be received oral antimicrobials (antibiotics, antifungals, antivirals or antiparasitics) within two months prior to the specimen collection.
5. Subjects must not consume large quantity of the commercial probiotics (> 10^8 cfu/day) and/or prebiotics. The foods with probiotic component (e.g. yogurts, fermented food, etc.) are acceptable.
6. Subjects must not have a major surgical operation of gastrointestinal tracts within five years prior to specimen collection. Appendectomy is acceptable.
7. Subjects must not have a history of uncontrolled gastrointestinal disorders, including
    - Inflammatory bowel diseases (IBD): ulcerative colitis (mild-moderate-severe) and Crohn's disease (mild-moderate-severe)
    - Irritable bowel syndrome (IBS) (moderate-severe)
    - Persistent infectious gastroenteritis, colitis or gastritis
    - Persistent or chronic diarrhea of unknown etiology
    - Persistent or chronic constipation
    - *Clostridium difficile* infection (It is not necessary to check. Only the case that

subject already diagnosed.)

- Untreated Helicobacter pylori infection (It is not necessary to check. Only the case that subject already diagnosed.)

8. Subject must not have intensive diabetes and/or obesity therapies.
9. Subjects with rapid weight change are not acceptable (either gain or lose > 5% of weight within three months).
10. Only subjects who have stayed in Yogyakarta city for longer than three years are acceptable.
11. Subject information is required to be collected as followed:

- Age
- Gender (must be male)
- Height
- Weight and weight change in the past three month
- Fecal frequency and defecation condition
- Allergy
- Medication
- Diseases
- Daily exercise
- Smoking and drinking
- Food frequency questionnaire or dietary records (dietary information is used to estimate energy intake from each nutrient.)
- Medical record (at least H1bAc or Blood glucose)

DNA extraction

Bacterial DNA was extracted from fecal samples by using the bead-beating method and subsequently purified as described previously with modification [1]. Stool sample was diluted 10-fold with RNAlater^{TM} (Invitrogen, Thermo Fisher Scientific, Vilnius, Lithuania) and homogenized by vortex. Then, 200 µl of the fecal sample diluents were mixed with 1 ml PBS and vortexed. After centrifugation at 20,000 × g for 5 min at 4 ºC, the supernatant was removed and washed twice with 1 ml of PBS buffer. The supernatant was discarded and the pellet was stored at -30 ºC until use. Three hundred milligram of glass beads (diameter, 0.1 mm) (TOMY SEIKO, Tokyo, Japan), 300 µl of Tris-SDS solution and 500 µl of TE buffer-saturated phenol (Wako Chemicals, Osaka, Japan) were added to the sample, and then shook vigorously by using a FastPrep® FP120 Cell Disrupter (Bio101 ThermoSavant, Waltham, MA, USA) at a speed of 5.0 m/sec for 30 s. Supernatant was collected by centrifugation at 20,000 × g for 5 min at 4 ºC. Four hundred microliter of phenol/chloroform/isoamyl alcohol (25:24:1 v/v; Wako Chemicals, Osaka, Japan) was added to the supernatant and shook vigorously with the use of the FastPrep® at a speed of 4.0 m/sec for 45 s. After centrifugation under the same condition as previous, a 250 µl of the supernatant was mixed with 25 µl of 3 M sodium acetate (pH 5.2; Sigma Aldrich, USA) and 300 µl

conc. isopropanol (Nacalai Tesque, Kyoto, Japan) was added. The DNA sample was precipitated at -30ºC for 30 min and then centrifuged under the same condition as previous to collect DNA pellet. The pellet of DNA was washed once by 500 µl cold 70% (v/v) ethanol (Wako Chemicals, Osaka, Japan) and air dried prior to suspension in 20 µl TE buffer (pH 8.0) and stored at -30ºC until use.

Sequence data process

The sequence data was processed by using the UPARSE pipeline consisting of following steps in USEARCH v9.2.64 software (http://drive5.com/usearch/download.html: accessed August 19th, 2020) [2]. (i) Pairs of the raw sequence reads were merged by using the fastq_mergepairs script with mismatched windows up to 10 bases. (ii) High quality sequences were selected from the merged sequences by using the fastq_filter script with an expected error score lower than 1.0. (iii) Dereplication was performed to find the unique sequences by using the derep_fulllength script. (iv) The obtained high-quality sequences were clustered into operational taxonomic units (OTUs) with 97% sequence identity by using the cluster_otus script while removing singleton OTUs. Also, PCR-chimera like sequences

were removed using the UCHIME algorithm [3]. As a result, 643 non-singleton OTUs were obtained. (v) The taxonomy of each OTU was identified with cut-off values higher than 0.8 in SINTAX algorithm [4] with the RDP training set v16. (vi) The taxonomic composition of each sample was estimated based on the OTU counts and OTU taxonomy by using the QIIME summarize_taxa_througy_plots.py command in QIIME version 1.9.1 (http://qiime.org/: accessed August 19th, 2020) [5].

Reference

1. Matsuki, T.; Watanabe, K.; Fujimoto, J.; Takada, T.; Tanaka, R. Use of 16s rRNA gene-targeted group-specific primers for real-time PCR analysis of predominant bacteria in human feces. *Appl Environ Microbiol.* **2004**, 70, 7220-7228, https://doi.org/10.1128/AEM.70.12.7220-7228.2004.

2. Edgar, R.C. Uparse: highly accurate OTU sequences from microbial amplicon reads. *Nat Methods* **2013**, 10, 996-998, https://doi.org/10.1038/nmeth.2604.

3. Edgar, R.C; Hass, B.J; Clemente, J.C; Quince, C.; Knight, R. UCHIME improves sensitivity and speed of chimera detection. *J Bioinform*. **2011**, 27(16), 2194-2200, https://doi.org/10.1093/bioinformatics/btr381.

4. Edgar, R.C. SINTAX: a simple non-Bayesian taxonomy classifier for 16S and ITS sequences. *bioRxiv.* **2016**, https://doi.org/10.1101/074161.

5. Kuczynski, J.; Stombaugh, J.; Walters, W.A; González, A.; Caporaso, G.J; Knight, R. Using QIIME to analyze 16S rRNA gene sequences from Microbial Communities. *Curr*

Protoc Bioinforma. **2011**, 10, 1-7, https://doi.org/10.1002/0471250953.bi1007s36.
